# Linguistic markers of emotional reactivity and their association with anxiety, depression, and stress among emergency call takers and dispatchers

**DOI:** 10.1371/journal.pone.0350551

**Published:** 2026-07-08

**Authors:** Vivian P. Ta-Johnson, Isabella M. Swafford, Paige Lindsey, Liliane D. Efinger, Janelle Kohler, Michael Fernandez, Victoria A. Torres, Suzy B. Gulliver, Sandra B. Morissette

**Affiliations:** 1 Department of Psychology, The University of Texas at San Antonio, San Antonio, Texas, United States of America; 2 San Antonio Police Department, Communications Division, San Antonio, Texas, United States of America; 3 Warriors Research Institute, Baylor Scott and White Health, Waco, Texas, United States of America; 4 Texas A&M, Health Science Center, College of Medicine, Bryan, Texas, United States of America; 5 Baylor College of Medicine, Houston, Texas, United States of America; University of Lahore - Raiwind Road Campus: The University of Lahore, PAKISTAN

## Abstract

Emergency call takers and dispatchers (ECDs) are routinely exposed to emotionally intense and highly stressful events, placing them at elevated risk for mental health challenges. Despite their critical role in emergency response, ECDs remain underrepresented in occupational mental health research, and it is unclear why some adapt more effectively than others under chronic stress. Cognitive appraisal theory suggests that differences in emotional reactivity—how individuals evaluate stimuli in terms of arousal and valence—may contribute to this variation. To test this, a sample of ECDs provided open-ended narratives of a stressful work-related event and completed the Depression, Anxiety, and Stress Scale (DASS-21). Narratives were analyzed using natural language processing to quantify emotional reactivity through the usage of high and low arousal language, and positively and negatively valenced language. Analyses revealed that greater use of negatively valenced language significantly predicted higher depression and anxiety, whereas positively valenced and arousal language did not. Stress symptoms were not significantly predicted by any linguistic markers of emotional reactivity. Follow-up analyses showed that individuals with moderate or higher depression and anxiety used significantly more negatively valenced language than those below threshold. These findings suggest that negatively valenced language serves as a robust marker of psychological distress among ECDs, even when narratives are elicited in contexts that naturally evoke negative emotion. Results highlight the potential of language-based tools for early, unobtrusive detection of distress, offering scalable approaches to promote resilience and support well-being in this critical workforce.

## Introduction

Emergency call takers and dispatchers (ECDs) are routinely exposed to emotionally intense and highly stressful events, placing them at elevated risk for mental health challenges [1–4]. As the first point of contact in many emergencies, ECDs receive high volumes of calls involving violence, injury, death, and threats to life, while simultaneously coordinating resources under time pressure and uncertainty. Unlike many first responders, ECDs’ exposure is often continuous and indirect, occurring through repeated verbal accounts of crises and ongoing radio traffic across shifts. Through these phone and radio communications, they are repeatedly immersed in high-stakes, emotionally charged events, and this cumulative exposure may contribute to chronic occupational stress. Despite their critical role in the emergency response system, they remain underrepresented in occupational mental health research compared to other first responder groups, such as police officers, emergency medical services (EMS) personnel, and firefighters [3].

This gap has scholarly and practical consequences. In many jurisdictions, ECDs’ institutional classification and uneven recognition as first responders shape access to funding streams, occupational protections, and dedicated wellness infrastructure, which in turn limits the systematic monitoring and support available for this workforce [5,6]. When a high-exposure group is simultaneously understudied and inconsistently resourced, the result is a weak evidence base for prevention and intervention, leaving key questions about risk and resilience unanswered. As a result, the factors that promote mental health and well-being among ECDs remain poorly understood. Particularly, it remains unclear why some ECDs adapt more effectively than others in the face of chronic occupational stress and exposure to potentially traumatic events (PTE; i.e., exposure to events involving threat to life, physical injury, or sexual violence) [7]. Cognitive appraisal theory provides a useful framework for addressing this question and posits that an individual’s subjective evaluation of a stressor (rather than the stressor itself) shapes emotional and behavioral outcomes [8]. Accordingly, individual differences in emotional reactivity, particularly the intensity and valence with which events are interpreted, may help explain variation in mental health outcomes among ECDs.

This framework also clarifies why arousal- and valence-related language should be informative. Appraisals involve evaluating an event’s meaning for the self (e.g., threat vs. safety, harm vs. benefit, controllability, and coping potential), and these evaluations generate affective responses that are commonly organized along two core dimensions: valence (negative to positive) and arousal (low to high) [8,9,10]. When ECDs describe a stressful incident in narrative form, they necessarily encode these appraisals through word choice, including the emotional tone of the description (e.g., negatively vs. positively valenced terms) and the intensity implied by the language used (e.g., high- vs. low-arousal terms). Thus, linguistic markers of valence and arousal provide a tractable, behavior-based index of appraisal-related emotional reactivity that can be quantified using natural language processing (NLP) and examined as a predictor of depression, anxiety, and stress.

Within the limited extant research on ECDs, studies have largely relied on using self-report symptom measures of mental health which are limited in their ability to capture the nuanced differences in how ECDs emotionally evaluate and respond to occupational stressors and PTEs. This limitation is critical as previous research suggests that emotional reactivity plays a key role in the development and persistence of mental health conditions [11,12]. NLP methods address these shortcomings by enabling researchers to gain psychological insights through the systematic analysis of verbal behavior, defined as the observable linguistic output people produce in speech or writing that can be quantified from text [13]. Patterns in language and verbal behavior reflect underlying cognitive and emotional processes and shed light on how individuals interpret and engage with their social environments. Research has used verbal behaviors to predict psychological characteristics such as distress and well-being [14], revealing that linguistic patterns are linked to mental health symptoms, coping strategies, and emotional regulation [13]. Although NLP has been increasingly applied across clinical and occupational domains to study psychopathology, it has never been applied to examine mental health and well-being among ECDs.

To address this gap, open-ended narratives were collected from a sample of ECDs describing a particularly stressful work-related event. NLP techniques were applied to quantify core dimensions of emotional reactivity, which in turn were used to predict depression, anxiety, and stress. These data have the potential to identify early verbal markers of psychological distress, which could serve as critical warning signs of more severe mental health challenges. By detecting indicators of emotional reactivity through language, this research can inform the future development of proactive monitoring systems aimed at identifying distress before it escalates. Furthermore, leveraging NLP opens new avenues for creating scalable, language-based mental health interventions tailored to the specific psychological demands of ECDs. Such developments could play a pivotal role in reducing burnout, promoting resilience, and supporting long-term well-being within this high-risk and often underrecognized workforce.

### Background literature

#### Mental health among ECDs and emotional reactivity.

ECDs serve as critical frontline personnel within emergency response systems and often act as the first point of intervention during crises [2]. ECDs are responsible for answering incoming calls, collecting essential information to initiate an emergency response, prioritizing responses, and dispatching emergency responders (including police, fire, and EMS) based on available information and assets. Importantly, ECDs must also gather real-time updates from first responders already engaged in active emergencies, such as officer-involved shootings that may evolve from traffic stops or foot pursuits, which requires them to assess public safety risks (e.g., speed, road conditions, pedestrian traffic) based on fragmented information coming from both distressed callers and on-scene personnel. Throughout these high-pressure situations, ECDs must remain calm, composed, and in control of both their call and their radio channel. Together, ECDs function as a vital link between distressed callers and first responders in the field to ensure that emergency assistance is delivered promptly and effectively.

The nature of working as an ECD involves managing emotionally charged and complex situations [15]. These demands frequently elicit psychological responses such as fear, helplessness, guilt, and persistent rumination [16]. ECDs are at heightened risk for a variety of mental health issues, including anxiety and depression [17], trauma-related symptoms [18], and suicidal ideation [19]. These risks are compounded by occupational stressors such as the need for rapid decision-making under uncertainty, exposure to abusive callers, insufficient training, and inconsistent supervision [2,20]. The cumulative effect often leads to emotional exhaustion, burnout [21], and physical health consequences such as sleep disturbances [17,22], cardiometabolic problems [23], and increased substance use [24]. Despite these challenges, most ECDs demonstrate notable resilience [17,25]. Protective factors such as peer support [26,27], organizational culture, and personal fulfillment from helping others [28] can buffer against distress. Experiences of post-traumatic growth and enhanced emotional competence have also been reported [2,29], suggesting that adaptive meaning-making processes may mitigate the impact of chronic stress.

The factors that contribute to risk for depression, anxiety, and stress under sustained occupational stress among ECDs remain unclear due to limited research and over-reliance on self-report mental health measures. According to cognitive appraisal theory [8], the experience of a stressor differs significantly between individuals depending on how they react and respond to the stressor. In other words, how ECDs experience occupational stressors is driven by their subjective interpretations of them rather than the stressor itself. This suggests that individual differences in emotional reactivity influence mental health outcomes and may help explain why some ECDs are more resilient than others under similar chronic occupational stress and PTE exposure [30,31].

Emotional reactivity is typically conceptualized along two primary dimensions: arousal, which reflects the degree of physiological activation or emotional intensity (i.e., calm or low arousal vs. exciting or high arousal), and valence, which captures the emotional quality of the response (i.e., positive valence vs. negative valence) [9]. These dimensions form the foundation of widely used models of emotion such as the Circumplex Model of Affect which posits that emotions can be represented within a two-dimensional space defined by arousal and valence [10]. Research shows that individual differences in emotional reactivity significantly influence psychological and physiological functioning. High emotional reactivity (marked by high arousal and negative valence) is linked to emotion regulation difficulties [32], impaired social functioning [33], and greater internalization of psychological distress [34]. In high-stress occupations such as emergency call taking and dispatching, elevated emotional reactivity can compromise decision-making and executive function [35,36], increase impulsivity [37], and facilitate threat-biased judgments [38]. Physiologically, heightened emotional reactivity is associated with increased autonomic arousal [39], disrupted sleep [40], and prolonged recovery from stress [41]. Heightened emotional reactivity is also linked with an elevated risk for depression, anxiety, and PTSD [42–44], while positive valence and moderate arousal are linked with greater resilience and more favorable treatment outcomes [45].

#### Language and mental health.

Language use is strongly related to mental health, both as a reflection of mental states and as a tool for assessing psychological well-being. For instance, depression is consistently linked to increased use of first-person singular pronouns and negative emotion words (e.g., “sad,” “hopeless”), reflecting heightened self-focus and rumination [46]. It is also associated with greater use of disfluencies and tentative language, suggesting diminished self-confidence and impaired cognitive processing [14,47]. Similarly, anxiety is linked with increased use of negative emotion words, affective language, absolutist terms (e.g., “always,” “never”), negations, and present-focused expressions [46,48,49]. Individuals with anxiety tend to use more socially oriented language but fewer positive emotion words, future-oriented terms, and produce shorter narratives, pointing to hypervigilance, social apprehension, and cognitive overload [49–52]. Stress-related language mirrors patterns seen in both depression and anxiety, including increased use of negative emotion words, disfluencies, and references to health and death [53–55]. High stress is further associated with language that is more self-focused, rigid, and uncertain, while lower stress is associated with greater use of positive emotion words and socially affiliative language [56–58].

Because narratives reflect not only what occurred but also how individuals evaluate and make meaning of events, linguistic patterns can serve as behavioral traces of appraisal processes. Appraisal-related emotional responses are expected to be expressed in language along core affective dimensions, including valence (negative to positive) and arousal (low to high), which directly align with the linguistic markers examined in the present study. Consistent with these patterns, prior work has increasingly applied NLP to quantify affective and distress-related signals in naturalistic text and demonstrate that language can index psychological risk and well-being. In clinical and mental health settings, NLP applied to unstructured text (e.g., clinical notes or crisis messages) has been used to identify markers of elevated risk, including suicidality and acute crisis states [59]. Similar approaches have been used in occupational contexts to capture stress- and burnout-relevant language, including analyses of healthcare worker discourse and burnout-related text signals [60]. Overall, this literature supports the premise that affective dimensions expressed in language can be quantified and linked to mental health outcomes, and it highlights the value of transparent, construct-linked measures when interpretability and privacy constraints are central. Building on this work, the present study extends affective language analysis to ECD narratives, a high-stress occupational group that has been largely absent from NLP-based occupational mental health research.

### The current study

The current study examined whether emotional reactivity, as expressed in language, predicts distress among ECDs. Specifically, the study tested whether arousal- and valence-related language in ECDs’ narratives of a particularly stressful work-related event predicts self-reported depression, anxiety, and stress. ECDs provided open-ended narratives recounting a particularly stressful work-related event and completed assessments that captured the severity of their depression, anxiety, and stress. Because narratives capture not only the events individuals experience but also how they interpret and find meaning in them [61], analyzing the language within these accounts offers an ecologically valid approach to assessing emotional reactivity and its connection to mental health outcomes.

To quantify emotional reactivity in language, the study used a dictionary-based NLP approach grounded in Warriner et al.’s [62] affective norms, which provide normative valence and arousal ratings for approximately 14,000 English words. For each narrative, the proportions of high versus low arousal terms and positive versus negative valence terms were computed, and these language measures were modeled as predictors of depression, anxiety, and stress. Based on the available research, it was hypothesized that greater use of high arousal language and negatively valenced language in narratives would significantly predict higher levels of anxiety, depression, and stress. Conversely, it was hypothesized that greater use of low arousal language and positively valenced language would significantly predict lower levels of these mental health outcomes.

This dictionary-based approach operationalizes emotional reactivity in a transparent, theory-aligned manner and is well-suited for ECD narratives because it yields interpretable, construct-linked language measures and does not require a large labeled training corpus as language features are computed directly from an established lexicon. It is also compatible with privacy constraints that limit data sharing given that the dictionaries, code, and derived outputs can be shared to support reproducibility without publicly releasing raw narratives. Using validated lexical norms also supports comparability across studies by anchoring language measures to established arousal and valence dimensions.

## Method

### Participants

Participants were full-time ECD employees within a large metropolitan police department. Due to the size and scope of the department, call takers and dispatchers function in separate job roles. Call takers are responsible for answering incoming emergency calls, non-emergency calls, and administrative calls, and collecting essential information to initiate an appropriate response. Dispatchers coordinate and prioritize the deployment of emergency responders, including police, fire, and EMS, based on this information provided by call takers. Dispatchers also monitor emergency responders to ensure their safety and aid in fulfilling request for additional resources or tools. All participants were required to be at least 18 years of age and currently employed as a call taker, dispatcher, or supervisor of either role.

The initial sample size consisted of *N* = 129 participants. Data from participants who either did not respond to the open-ended question or provided “n/a” as their response were excluded from analysis (*n* = 19). In addition, responses containing very low word counts (fewer than five words) were also excluded from analysis (*n* = 4). Very short responses often lack useful signals and are typically removed from analysis due to limited syntactic and semantic content [63]. As such, the final sample included *N* = 106 participants consisting of 51.89% call takers, 42.45% dispatchers, and 5.66% other (i.e., individuals who had both call-taker and dispatcher roles). On average, participants had 72.95 months (*SD* = 87.35) of employment. Ages ranged from 21 to 71 (*M* = 34.59 years, *SD* = 10.87 years) with 77.14% identifying as female. Race and ethnicity were assessed using single-choice (non-multi-select) items. For race, 89.62% identified as White, 3.77% as Black or African American, and 6.60% as Other. For ethnicity, 68.87% identified as Hispanic or Latino. Demographic characteristics for the initial sample can be found in S1 Table.

### Procedure

Institutional Review Board (IRB) approval was secured prior to initiating any study procedures. ECDs were recruited by study personnel and agency leadership between October 1 and December 31 of 2024 using flyers and emails inviting them to take part in an online study about their work experiences and well-being. Participants were instructed to either scan the QR code provided on recruitment flyers or click on the link included in email invitations if they were interested in the study. The QR code and link directed participants to a Qualtrics survey where they were presented with a detailed electronic study information sheet, including a description of the study’s purpose and voluntary nature, procedures, assurances regarding data privacy, data protections, confidentiality, and contact information for any questions and concerns. Individuals who did not meet study requirements or did not consent were instructed to exit the survey. For those who chose to proceed, informed consent was obtained through an implied-consent process in which continuing beyond the consent page indicated agreement to participate. No written or verbal consent was collected in accordance with the study’s protocol approved by the IRB. Individuals provided demographic information, completed the Depression, Anxiety, and Stress Scale (DASS-21) [64], answered an open-ended question about a stressful situation they recently experienced while at work, and several additional measures that were not pertinent and were thus excluded from analysis. After completing the survey, participants were directed to a separate website so they could opt to provide their contact information to receive compensation in the form of a $75 Amazon gift card.

The data underlying this study contain sensitive information about participants’ mental health and occupational experiences and are subject to restrictions imposed by the IRB at the University of Texas at San Antonio. Participants did not provide consent for data sharing, and the IRB-approved protocol limits access to authorized members of the research team. As such, the dataset cannot be made publicly available, and no participant-level data can be shared. Requests for access may be directed to the UTSA IRB (irb@utsa.edu) and will be reviewed in accordance with IRB requirements and applicable data-use protections. To support transparency, the code used for data analysis dictionaries, and documentation can be found at the following link: https://doi.org/10.17605/OSF.IO/FSV23..

### Measures

#### Demographics.

Demographic information was assessed to characterize the sample, including their gender, age, race, ethnicity, employment classification (full-time or part-time), role (call taker, dispatcher, dispatcher with prior call-taker experience, supervisor call taker, supervisor dispatcher, other), and length of employment (in months).

#### Depression, Anxiety, and Stress Scale (DASS-21).

The DASS-21 contains 21 items that measure emotional states of anxiety, depression, and stress [64]. Participants were instructed to read each item and indicate the extent to which the item applied to them over the past week. Items are scored on a 4-point Likert-type scale using scale alternatives ranging from “Did not apply to me at all” to “Applied to me very much or most of the time”. In the final sample, depression (Cronbach’s α = 0.91) demonstrated excellent internal consistency, and anxiety (Cronbach’s α = 0.80) and stress (Cronbach’s α = 0.87) demonstrated very good internal consistency.

#### Open-ended question.

Participants were prompted to answer the following question in free response format by typing in the provided textbox: “Throughout your job, you may have faced situations that have caused you stress. For this question, please describe a high-stress situation you experienced while working as a call taker or dispatcher. Please include as many details as possible about the situation as well as the feelings you had at the time.” Participants were not subject to any limitations on response length and were permitted to provide as much detail as they desired. Responses averaged 122.51 words (Median _word count_ = 87.50, *SD*_word count_ = 114.71, minimum _word count_ = 10, maximum _word count_ = 620) and correlations with study variables are reported in S3 Table.

#### Analytic strategy.

All narratives were first preprocessed using standard text-cleaning procedures. Specifically, all text was lowercased, and non-alphabetic characters (excluding numbers and punctuation) were removed. Lemmatization or stemming was not applied. Each response was then scored on high arousal, low arousal, negative valence, and positive valence using Warriner et al.’s [62] database of emotion norms. This database includes ~14,000 English terms reliably scored on valence and arousal, and was used to construct four dictionaries containing terms scoring high on arousal, low on arousal, high on valence (positively valenced), and low on valence (negatively valenced). Similar to previous research [e.g., 65], terms that scored at least one standard deviation above and below the average valence score were labeled as high and low on valence, respectively, and terms that scored at least one standard deviation above and below the average arousal score were labeled as high and low on arousal, respectively. High valence and high arousal terms are more positive and intense, respectively, and low valence and low arousal terms are more negative and calmer, respectively. Table 1 reports sample terms scoring high and low on arousal and valence. The dictionaries were then used to detect the percentage of terms in each response that scored high and low in valence and arousal (which is standard in dictionary-based NLP to minimize confounding by verbosity [66]) using the *quanteda* package in R [67]. This yielded a high arousal score, a low arousal score, a high valence score, and a low valence score (4 individual scores) for each response. Higher scores indicated greater usage of the given emotional reactivity measure. Consistent with standard dictionary-based approaches, negation handling was not implemented in the present analysis to preserve interpretability and comparability of dictionary-based measures [68]. Anxiety, depression, and stress scores were calculated for each individual by summing the responses to the items within each respective subscale. Prior to scoring, the data were screened for missing values, and no missing data were identified. These total subscale scores were then used in subsequent analyses.

**Table 1:** Example terms scoring high and low on arousal and valence.

High Arousal	*amazing, kill, euphoria, venom, scream*
Low Arousal	*geriatric, float, book, recline, apartment*
High (Positive) Valence	*attractive, clean, protect, truth, live*
Low (Negative) Valence	*blood, danger, hate, rude, shoot*

## Results

Given the modest sample size, participant role (call taker, dispatcher, other), gender (male, female), age, and employment length (in months) were considered as potential control variables. Only variables demonstrating significant associations with the outcome variables were included in subsequent multiple regression models to maintain model parsimony and minimize overfitting. ANOVAs were conducted to determine if anxiety, depression, and stress levels significantly differed across participant role and gender. Differences across participant role were not observed for depression, *F*(2, 103) = 0.48, *p* = .62, anxiety, *F*(2, 103) = 0.18, *p* = .83, or stress, *F*(2, 103) = 0.25, *p* = .78. Gender differences were not observed for depression, *F*(1, 103) = 0.95, *p* = .33, anxiety, *F*(1, 103) = 1.26, *p* = .26, or stress, *F*(1, 103) = 1.90, *p* = .17. Zero-order correlations were also conducted to determine if depression, anxiety, and stress levels were significantly related to age and employment length. Age was not correlated with depression, *r*(104) = −0.03, *p* = .75, anxiety, *r*(104) = −0.04, *p* = .69, or stress, *r*(104) = −0.05, *p* = .62. Employment length was also not correlated with depression, *r*(104) = 0.08, *p* = .36, anxiety, *r*(104) = 0.12, *p* = .24, or stress, *r*(104) = 0.04, *p* = .67. As such, none of the potential control variables were entered into subsequent multiple regression models.

Descriptive statistics and zero-order correlations across all predictor and outcome variables are reported in Table 2. Depression and anxiety were strongly correlated, and both also showed strong correlations with stress. Despite these associations, the constructs remain theoretically distinct and are assessed as separate subscales within the DASS-21, supporting their treatment as independent outcomes in the predictive analyses.

**Table 2:** Descriptive statistics and zero-order correlations of primary predictors and outcome variables.

	M(SD)	HiA	LoA	V+	V-	Anx	Dep	Str
HiA	3.87(2.55)	--						
LoA	5.17(2.78)	−0.08	--					
V+	6.80(3.49)	0.03	0.02	--				
V-	4.19(3.24)	0.53***	−0.05	−0.07	--			
Anx	7.04(7.04)	0.15	0.07	0.08	0.34***	--		
Dep	8.15(8.78)	0.09	−0.01	0.10	0.31**	0.64***	--	
Str	12.28(8.56)	0.02	0.09	0.13	0.13	0.77***	0.73***	--

*Note.* M = mean; SD = standard deviation; HiA = High Arousal; LoA = Low Arousal; V+ = Positive Valence; V- = Negative Valence; Anx = Anxiety; Dep = Depression; Str = Stress; **p < .01; ***p < .001.

Based on DASS-21 cutoff scores the final sample exhibited depressive symptoms within the normal range on average, with average mild levels of anxiety and stress. Additionally, 25.47% of participants reported moderate to extremely severe levels of depressive symptoms, 31.13% reported moderate to extremely severe levels of anxiety, and 20.76% reported moderate to extremely severe levels of stress. Further details regarding the initial and final samples’ DASS-21 cutoff scores are provided in S4 Table.

Three multiple regression models were conducted to examine whether response scores on high and low levels of arousal and positive and negative valence significantly predicted depression, anxiety, and stress. Model assumptions (linearity, residual normality, homoscedasticity, and multicollinearity) were evaluated using standard diagnostic procedures. Scatterplots of predictors against each outcome indicated no meaningful departures from linearity. Residual Q-Q plots suggested approximate normality, and residuals versus fitted plots indicated roughly constant variance. No influential observations were identified (all Cook’s D < 1). Variance inflation factors were uniformly low (all < 2), providing no indication of problematic multicollinearity.

Full model results are reported in Table 3. The overall model predicting depression was significant, *F*(4, 101) = 3.45, *p* = 0.01, *R*^2^ = 0.12. Negatively valenced language was a significant predictor, such that greater use of negatively valenced language significantly predicted higher levels of depressive symptoms. High arousal language, low arousal language, and positively valenced language were not significant predictors of depressive symptoms. Next, the overall model predicting anxiety was significant, *F*(4, 101) = 3.87, *p* = 0.01, *R*^2^ = 0.12. Negatively valenced language was a significant predictor such that greater use of negatively valenced language significantly predicted higher levels of anxiety. High arousal language, low arousal language, and positively valenced language were not significant predictors of anxiety. Last, the overall model predicting stress was not significant, *F*(4, 101) = 1.30, *p* = 0.28, *R*^2^ = 0.05. High arousal language, low arousal language, positively valenced language, and negatively valenced language were not significant predictors of stress. Post hoc power analyses conducted in G*Power (Faul et al., 2009) indicated that achieved power to detect the observed effects was .87 for the depression model, .89 for the anxiety model, and .40 for the stress model. To assess the robustness of these findings, additional regression models were estimated including age, gender, and employment length as covariates. The pattern of results remained unchanged, and full results are reported in S3 Table.

**Table 3:** Full results of multiple regression models.

	Anx	Dep	Str
Predictors	β(SE), 95% CI	β(SE), 95% CI	β(SE), 95% CI
HiA	−0.12(0.30), [−0.72, 0.49]	−0.41(0.38), [−1.17, 0.35]	−0.26(0.39), [−1.03, 0.51]
LoA	0.21(0.24), [−0.25, 0.68]	0.01(0.30), [−0.60, 0.58]	0.28(0.30), [−0.31, 0.88]
V+	0.20(0.19), [−0.17, 0.58]	0.34(0.24), [−0.13, 0.81]	0.36(0.24), [−0.12, 0.83]
V-	0.81(0.24), [0.33, 1.28]**	1.03(0.30), [0.43, 1.62]***	0.48(0.30), [−0.13, 1.08]

β = standardized regression coefficient; SE = standard error; HiA = High Arousal; LoA = Low Arousal; V+ = Positive Valence; V- = Negative Valence; Anx = Anxiety; Dep = Depression; Str = Stress; **p < .01; ***p < .001.

As a follow-up to address multiple testing, the false discovery rate (FDR) was controlled using the Benjamini-Hochberg (BH) procedure separately within each model with four predictor tests per model. After FDR correction, negative valence remained a significant predictor of depression (BH-adjusted *p* ≤ .004) and anxiety (BH-adjusted *p* = .004). No predictors significantly predicted stress (BH-adjusted *p*s ≥ .27).

To complement the dimensional analyses, the average percentage of negatively valenced words was compared between narratives written by participants who met clinical cutoffs for moderate or higher depression and anxiety and those written by participants who scored below threshold. Severity thresholds were applied to the corrected (×2) DASS-21 scores. Welch’s *t* tests revealed that individuals with moderate or higher depressive symptoms used significantly more negatively valenced terms (*M* = 5.77, *SE* = 0.92) than those below threshold (*M* = 3.65, *SE* = 0.26), *t*(30.23) = 2.22, *p* = .03, Glass’s Δ = 0.92, 95% CI [0.23, 1.12]. Similarly, participants who exhibited moderate or higher anxiety used significantly more negatively valenced terms (*M* = 5.60, *SE* = 0.77) than those below threshold (*M* = 3.55, *SE* = 0.27), *t*(40.07) = 2.52, *p* = .02, Glass’s Δ = 0.89, 95% CI [0.24, 1.07]. To address multiple testing across the two Welch’s *t* tests, *p* values were adjusted using the BH procedure, treating the two tests as a single family to control the FDR. Both comparisons remained significant after adjustment (BH-adjusted *p*s = .03).

These findings indicate that higher use of negatively valenced language is associated with higher depression and anxiety and differentiates participants above versus below clinical risk thresholds in this sample. It is important to note that threshold-based comparisons are intended as a descriptive complement to the continuous analyses and should not be interpreted as validating negative valence as a clinical screening tool. Any interpretation of negative valence as a potential indicator of clinical risk is preliminary and requires independent validation against clinician-rated outcomes and prospective prediction.

## Discussion

ECDs are repeatedly exposed to emotionally intense situations frequently involving PTEs, placing them at an elevated risk for developing mental health problems such as anxiety, depression, and stress-related disorders. Although many ECDs demonstrate mental well-being in response to high-stress exposure, it is imperative to identify novel risk factors for depression, anxiety, and stress, both for their personal well-being given that they play a critical role in public safety. Language is a meaningful behavioral marker of mental health distress, and NLP enables researchers to gain insight into the psychological processes that shape these outcomes through the quantitative analysis of verbal behavior. Guided by cognitive appraisal theory, NLP was used to quantify emotional reactivity in open-ended narratives from ECDs recounting particularly stressful work-related events. In turn, how these linguistic markers predicted depression, anxiety, and stress were examined.

The findings partially supported the study hypotheses. As expected, higher use of negatively valenced language significantly predicted higher levels of both depression and anxiety, which often co-occur. This finding aligns with prior research showing that increased use of negatively valenced language reliably signals psychological distress and internalizing symptoms across a diverse range of populations [46,48]. Follow-up analyses compared participants who met clinical cutoffs for moderate or higher levels of depression and anxiety to those who did not. Individuals above threshold used significantly more negatively valenced language in their narratives. From a cognitive appraisal perspective, greater negatively valenced language in narratives may reflect more negatively toned evaluations of stressful events, and this is consistent with research showing that affective characteristics of spoken and written narratives track emotional expression and psychological distress [69–71].

Counter to expectations, higher use of positively valenced language did not significantly predict lower depression, anxiety, or stress. This finding was particularly unexpected in relation to depression, which is often characterized by low positive affect [72]. However, Chiang and Bai [42] reported a similar pattern among parents and adolescents, suggesting that higher positive valence may be associated with reduced mental health symptom severityonly under certain contextual or individual conditions. Thus, under chronically high-stress contexts such as emergency call taking and dispatching, negatively valenced language may be more diagnostic of depression and anxiety than positively valenced language. This is consistent with evidence that sustained stress is associated with heightened threat sensitivity and stronger negative affect in response to negatively valanced stimuli—hallmarks of internalizing psychopathology [73,74]—and that negatively valenced emotional reactivity shows a stronger and more consistent association with emotional dysregulation and psychological distress than positively valenced reactivity [75]. Further, research in high-stress occupational environments indicate that negatively valenced responses are more consistently linked to psychological strain and mental health symptoms than the absence of positively valenced responses, which may be suppressed or contextually constrained under chronic stress [28,76]. In other words, ECDs may be less likely to exhibit positively valenced reactions at work, not necessarily due to poor mental health, but because the demands of the role limit its expression. Moreover, because positive and negative valence are distinct rather than opposing dimensions [77], they may be orthogonally related to mental health outcomes, which may explain why higher use of negatively valenced language, but not lower use of positively valenced language, predicted higher depression and anxiety.

Contrary to expectations, high- and low-arousal language use did not significantly predict symptoms of depression, anxiety, or stress. This null pattern should be interpreted cautiously, and several non-mutually exclusive explanations are plausible. Prior theory suggests that valence is more consistently and readily expressed in language than arousal, which may be more variable and context-dependent [10,78]. In retrospective narratives, speakers may prioritize evaluative meaning-making over describing momentary intensity, rendering language reflecting arousal less salient when recalling past experiences than during immediate responding [54,79]. Consistent with this, work on autobiographical and trauma narratives suggests that language reflecting valence can be more prominent than language reflecting arousal, particularly when accounts are temporally distant from the original experience [80]. Finally, occupational norms may shape expressive patterns. In ECD contexts, maintaining composure is emphasized as part of effective performance which could reduce explicit linguistic signaling of arousal in written accounts [81]. Importantly, the present data cannot distinguish among these mechanisms, and future work combining narratives with concurrent indices of arousal (e.g., physiological measures or time-anchored reports) is needed to test these explanations directly.

In addition, neither high nor low arousal language, nor negatively valenced or positively valenced language, significantly predicted stress. This finding was unexpected and may reflect the complexity and chronicity of stress within this occupational workforce. Individuals in high stress occupations often develop coping strategies or emotion regulation techniques that allow them to manage or compartmentalize stress [82,83]. A recent systematic review also reported that the primary adaptive response to stressful events among first responders was avoidance and disconnection [84]. This suggests that linguistic markers of emotional reactivity could be suppressed among ECDs, especially in written reflections, making emotionally reactive language a less sensitive indicator of stress compared to other linguistic features like cognitive complexity and self-referential language [58]. Moreover, because depression and anxiety diagnoses are highly comorbid [85], this overlap may have contributed to similar findings for depression and anxiety while attenuating unique associations with stress. Future work should consider modeling these outcomes simultaneously or using structural approaches that can partition shared versus distinct variance. Further, the regression model predicting stress explained relatively little variance in stress which corresponded to low achieved power (.40) to detect an effect of the observed magnitude and indicates limited sensitivity in this sample. Accordingly, the nonsignificant predictors for stress should be interpreted cautiously. Although the results are consistent with a null association, small effects cannot be ruled out. Future studies with larger samples are needed to more definitively evaluate linguistic predictors of stress.

Taken together, our findings highlight that greater use of negatively valenced language is a key indicator of psychological distress—specifically depression and anxiety—among ECDs when recalling a stressful work-related event. The absence of significant associations for high and low arousal language not only reinforces this conclusion, but also challenges prior assumptions that arousal is inherently tied to psychological distress and underscores the importance of considering occupational context when assessing emotional reactivity. Our findings also provide empirical support for using NLP to assess emotional reactivity in naturalistic language and demonstrate its potential to provide a complementary, behavior-based signal that can triangulate with self-report measures rather than replace them. Importantly, because outcomes in the present study were assessed via self-report and language features were derived from dictionary-based scoring, these findings should be interpreted as evidence of convergent associations rather than incremental validity beyond self-report. Establishing added predictive value will require validation against clinician-rated outcomes, behavioral/occupational indicators, or longitudinal change.

It is important to note that the open-ended narrative prompt asked participants to describe a particularly stressful work-related event. As such, the prompt may have implicitly encouraged the use of negatively valenced language, potentially introducing a baseline level of negative affect in participants’ responses and constrained variability in affective tone. However, the key insight from our findings is that even within this constrained context, individual differences in the extent of negative language use remained predictive of depression and anxiety. This pattern suggests that the signal lies not in whether negative language appears—which is expected given the prompt— but in how strongly it is expressed. At the same time, a stress-focused prompt may attenuate associations for positively valenced language (and potentially arousal language) and limits generalizability to narratives elicited by neutral or positive prompts. Future research should test whether these linguistic predictors replicate across a broader range of narrative prompts (e.g., neutral events, positive or helping experiences, or routine calls) and consider within-person designs that compare language patterns across event types.

The consistent link between greater use of negatively valenced language and higher levels of depression and anxiety suggests that language-based measures may help inform the development of early detection and intervention strategies within emergency call centers. For instance, brief written check-ins or prompted reflections (e.g., journaling about recent stressful calls) could be integrated into routine wellness protocols and analyzed via NLP to detect patterns of negatively valenced language and identify ECDs who may benefit from further support. This approach may offer a more unobtrusive and de-stigmatizing means of identifying those who may need additional support given NLP analyzes naturally occurring language data without relying on potentially intrusive questioning or self-report measures. This may be particularly important for first responders who may be hesitant to reach out for mental health support. The relative ease of integrating NLP into routine wellness protocols and its scalability suggest that it may be well-suited for the operational demands of emergency call-taking and dispatching environments [86]. NLP also has potential applications for improving emergency communication systems. For example, some ECD agencies now use call-handling software that automatically transcribes conversations and flags high-risk keywords (e.g., “gun,” “shooting,” “knife”) in real time [87]. Building on these systems, future work could evaluate whether integrating NLP to monitor both the content of callers’ communication and the language used by ECDs provides useful information for enhancing the well-being of ECDs, identifying cumulative stress exposure, and informing early intervention efforts.

The finding that participants who met clinical cutoffs for moderate or higher depression and anxiety used significantly more negatively valenced language than those below threshold offers a preliminary benchmark for identifying psychologically meaningful levels of negative valence. This may inform the development of NLP-based monitoring systems for detecting risk. For instance, brief written check-ins could be automatically screened for high levels of negatively valenced language to flag individuals who may benefit from further psychological support. This threshold-based approach represents a potentially ecologically valid and unobtrusive method for detecting distress, especially in occupational settings where stigma or logistical constraints may limit the use of traditional mental health assessments. While negatively valenced language differentiated participants above versus below DASS-21 subscale thresholds in this sample, this does not establish clinical utility. Future work is needed to test its predictive value and calibration in larger samples using longitudinal designs and external criteria.

Given that negatively valenced language emerged as a more reliable indicator of distress than arousal language or positively valenced language, these findings may help inform future work examining interventions that target negative cognitive-emotional processing such as cognitive restructuring, expressive writing, or acceptance and commitment-based approaches. Additionally, training programs or workshops that equip ECDs with skills to recognize and reframe negative thought patterns could help strengthen psychological resilience and promote long-term well-being.

This study focuses on an understudied yet critical population and, to our knowledge, is the first to use NLP to examine the relationship between emotional reactivity and mental health among ECDs. By using open-ended, unrestricted narrative responses, we enabled participants to express themselves freely and authentically, which generated rich, ecologically valid qualitative data ideal for assessing emotional reactivity through language. To analyze these narratives, a validated dictionary-based approach widely used in psychological and computational linguistics research was employed to quantify emotional reactivity in text. This method draws on a large and rigorously developed lexical database that provides reliable valence and arousal ratings for a broad range of words.

Compared to other NLP approaches (e.g., topic modeling), dictionary-based methods offer several advantages: they are extensively validated, applicable across diverse text corpora, and highly transparent. Importantly, they allow researchers to examine the exact words contributing to emotional reactivity scores which provides interpretability that is often lacking in more opaque computational models [88,89]. In addition, arousal and valence scores were calculated as the proportion of relevant terms in each response which ensured that the results were independent of overall narrative length.

Despite these strengths, several limitations warrant consideration. All participants were recruited from a single emergency communications center, which prevented us from accounting for department-level variables known to influence the well-being and emotional expression of ECDs such as organizational structure [2,15] and workplace culture [90]. Also, the use of a $75 Amazon gift card as an incentive may have introduced participation bias by attracting people more motivated by the reward. The modest sample size limited our ability to examine interaction effects between emotional reactivity dimensions and mental health outcomes. As a result, it remains unclear whether the effect of one emotional reactivity dimension on psychological distress depends on the level of the other emotional reactivity dimension.

Most participants identified as White (89.62%), Hispanic/Latino (68.87%), and female (77.14%). This demographic concentration limits generalizability to more racially and ethnically diverse ECD populations, although it also reflects key national workforce patterns in which ECDs are disproportionately female and increasingly Hispanic/Latino [1,91]. In this sense, the sample captures meaningful features of the contemporary ECD workforce rather than solely reflecting recruitment bias. At the same time, the overrepresentation of White participants underscores a broader limitation common in psychological research, where samples drawn from Western, educated, industrialized, rich, and democratic (WEIRD) contexts remain pervasive [92].

Demographic concentration may also shape linguistic norms and, in turn, the interpretation of dictionary-based scores. Lexical choices, emotional expression norms, and narrative style can vary across demographic groups, regional subcultures, and organizational environments, potentially influencing baseline rates of valence- and arousal-related terms independent of psychological distress. Accordingly, these findings may not generalize to agencies with different cultural or organizational communication norms. Future research should recruit larger, more diverse samples across multiple departments and examine whether linguistic predictors function similarly across groups (e.g., via measurement invariance or differential item functioning tests).

It is also important to recognize that many positively and negatively valenced terms are embedded in the occupational lexicon of ECDs and are reinforced through training and the situational demands of the job. Consequently, the presence of such words may reflect professional communication norms or incident content rather than the narrator’s underlying psychological state. This is especially relevant because narratives from ECDs may include job-specific or incident-related terminology that is rated as highly negative in affective norms but often functions as an operational descriptor. As a result, dictionary-based valence scores may partially capture case content or call-type mix, not only appraisal-related emotional reactivity. At the same time, the magnitude and patterning of valenced language may still carry psychological information. Even within a shared occupational vocabulary, disproportionately frequent negative language, or a more globally negative evaluative tone, may reflect heightened distress or depression and anxiety. Thus, the narratives in the present study likely reflect a mixture of role-driven language requirements and individual differences in expression as high-frequency operational or case-specific terms were not excluded from the current analysis. Future work should adopt domain-sensitive strategies to disentangle operational content from evaluative tone. For example, studies could preregister a reduced lexicon that excludes common operational terms (with a transparent rationale), separate incident-content terms from evaluative or emotion terms, and/or adjust for incident-content indicators (e.g., violence-related keywords or call categories). Future research should also use prompts that elicit a broader range of emotional contexts (e.g., neutral events, routine calls, or positive/helping experiences) and recruit larger, more diverse samples across agencies to more precisely identify when negatively valenced language reflects occupational case content versus distress-related appraisal. Similarly, because negation was not modeled in the present analysis, future research should incorporate methods that account for negation and other contextual features to improve the precision of language-based measures.

Although negatively valenced language significantly predicted depression and anxiety, the cross-sectional design of the study precludes any causal inferences. Longitudinal research is needed to determine whether shifts in emotionally reactive language precede or result from changes in mental health outcomes. The use of retrospective narratives introduces the possibility of recall biases, especially in accounts involving intense emotional experiences [93,94]. Future studies could mitigate this limitation by examining language use in real time through transcripts of communication or written reflections from ECDs collected immediately after stressful events.

Finally, due to IRB restrictions and the sensitive nature of participants’ mental health and occupational narratives, the underlying data cannot be made publicly available, which places some limits on full reproducibility using the original dataset. Access may be granted through IRB review under restricted conditions, and all analysis code, dictionaries, and documentation are publicly available on the OSF repository.

## Conclusion

This study highlights the value of linguistic markers, particularly negatively valenced language, as correlates of psychological distress among ECDs. Using a dictionary-based NLP approach, we found that greater negatively valenced language in ECDs’ stressful-event narratives was associated with higher depression and anxiety, whereas arousal language and positively valenced language were not reliably related to depression and anxiety. These findings underscore the potential utility of affective language features as a complementary, low-burden signal for screening and hypothesis generation, while also emphasizing the importance of context-sensitive interpretation in this occupational setting. Importantly, the present data are cross-sectional and do not permit causal inference or conclusions about temporal ordering. Longitudinal and real-time designs (e.g., repeated narratives, ecological momentary assessment, or linkage to objective wellness indicators) are needed to determine whether changes in language precede changes in distress, reflect concurrent distress, or both. Ultimately, this work advances our understanding of mental health in ECDs and lays a critical foundation for future research aimed at improving well-being in one of the most psychologically challenging frontline professions.

## Supporting information

S1 Table Demographic characteristics of initial sample (N = 129). (DOCX)

S2 Table DASS-21 cutoff scores. (DOCX)

S3 Table Correlations between response word count and primary study variables. (DOCX)

S4 Table Regression models predicting depression, anxiety, and stress with age, gender, and employment length as covariates. (DOCX)
