# Supplementary material for: Linguistic markers of emotional reactivity and their association with anxiety, depression, and stress among emergency call takers and dispatchers
Source: PLoS One. 2026 Jul 8;21(7):e0350551. doi: 10.1371/journal.pone.0350551 (PMC13345231; doi:10.1371/journal.pone.0350551)
Supplement: S1 Table — (DOCX) [file pone.0350551.s001.docx]

**S1 Table**

**Demographic Characteristics of Initial Sample (*N* = 129)**

| **Role** | | | | *n*(%) |  |
| --- | --- | --- | --- | --- | --- |
| Call Taker (includes call taker supervisors) | | | | 63(48.84%) |  |
| Dispatcher (includes dispatcher supervisors and dispatchers with prior call-taker experience) | | | | 58(44.96%) |  |
| Other (individuals with both call-taker and dispatcher roles) | | | | 8(6.20%) |  |
| **Race** | | | |  |  |
| Black | | | | 5(3.88%) |  |
| White | | | | 115(89.15%) |  |
| Other | | | | 9(6.98%) |  |
| **Ethnicity** | | | |  |  |
| Hispanic or Latino | | | | 89(68.99%) |  |
| Not Hispanic or Latino | | | | 40(31.01%) |  |
| **Gender** | | |  | | |
| Female | | | 66(66.67%) | | |
| Male | | | 32(32.32%) | | |
| Other | | | 1(1.01%) | | |
| **Employment Classification** | | |  | | |
| Full-time (40 or more hours per week) | | | 129(100%) | | |
|  | **Range** | ***M*(*SD*)** | | |  |
| **Employment (Months)** | 1 to 486 | 69.09(83.63) | | |  |
| **Age (Years)** | 20 to 71 | 33.97(10.78) | | |  |
| **Depression** | 0 to 34 | 8.19(8.95) | | |  |
| **Anxiety** | 0 to 28 | 7.13(7.07) | | |  |
| **Stress** | 0 to 38 | 12.16(8.75) | | |  |
