# Supplementary material for: Linguistic markers of emotional reactivity and their association with anxiety, depression, and stress among emergency call takers and dispatchers
Source: PLoS One. 2026 Jul 8;21(7):e0350551. doi: 10.1371/journal.pone.0350551 (PMC13345231; doi:10.1371/journal.pone.0350551)
Supplement: S2 Table — (DOCX) [file pone.0350551.s002.docx]

**S2 Table**

**DASS-21 Cutoff Scores**

| **Initial Sample** | | | |
| --- | --- | --- | --- |
|  | *n*(%) | | |
| **Cutoffs** | **Depression** | **Anxiety** | **Stress** |
| Normal | 87(67.44%) | 74(57.36%) | 86(66.67%) |
| Mild | 8(6.20%) | 14(10.85%) | 17(13.18%) |
| Moderate | 19(14.73%) | 20(15.50%) | 14(10.85%) |
| Severe | 7(5.43%) | 12(9.30%) | 9(6.98%) |
| Extremely Severe | 8(6.20%) | 9(6.98%) | 3(2.33%) |
| **Final Sample** | | | |
| Normal | 71(66.98%) | 60(56.60%) | 70(66.04%) |
| Mild | 8(7.55%) | 13(12.26%) | 14(13.21%) |
| Moderate | 16(15.09%) | 16(15.09%) | 13(12.26%) |
| Severe | 4(3.77%) | 10(9.43%) | 7(6.60%) |
| Extremely Severe | 7(6.60%) | 7(6.60%) | 2(1.89%) |
