# Supplementary material for: Linguistic markers of emotional reactivity and their association with anxiety, depression, and stress among emergency call takers and dispatchers
Source: PLoS One. 2026 Jul 8;21(7):e0350551. doi: 10.1371/journal.pone.0350551 (PMC13345231; doi:10.1371/journal.pone.0350551)
Supplement: S3 Table — (DOCX) [file pone.0350551.s003.docx]

**S3 Table**

**Correlations Between Response Word Count and Primary Study Variables**

|  | *M*(*SD*) | HiA | LoA | V+ | V- | Anx | Dep | Str | WC |
| --- | --- | --- | --- | --- | --- | --- | --- | --- | --- |
| HiA | 3.87(2.55) | -- |  |  |  |  |  |  |  |
| LoA | 5.17(2.78) | -0.08 | -- |  |  |  |  |  |  |
| V+ | 6.80(3.49) | 0.03 | 0.02 | -- |  |  |  |  |  |
| V- | 4.19(3.24) | 0.53*** | -0.05 | -0.07 | -- |  |  |  |  |
| Anx | 7.04(7.04) | 0.15 | 0.07 | 0.08 | 0.34*** | -- |  |  |  |
| Dep | 8.15(8.78) | 0.09 | -0.01 | 0.10 | 0.31** | 0.64*** | -- |  |  |
| Str | 12.28(8.56) | 0.02 | 0.09 | 0.13 | 0.13 | 0.77*** | 0.73*** | -- |  |
| WC | 122.51(114.71) | -0.11 | 0.03 | -0.10 | -0.26** | -0.02 | -0.07 | 0.07 | -- |
| *Note.* *M* = mean; *SD* = standard deviation; HiA = High Arousal; LoA = Low Arousal; V+ = Positive Valence; V- = Negative Valence; Anx = Anxiety; Dep = Depression; Str = Stress; WC = Word Count; ***p*< .01; ****p*< .001. | | | | | | | | | |
