# Supplementary material for: Linguistic markers of emotional reactivity and their association with anxiety, depression, and stress among emergency call takers and dispatchers
Source: PLoS One. 2026 Jul 8;21(7):e0350551. doi: 10.1371/journal.pone.0350551 (PMC13345231; doi:10.1371/journal.pone.0350551)
Supplement: S4 Table — (DOCX) [file pone.0350551.s004.docx]

**S4 Table**

**Regression Models Predicting Depression, Anxiety, and Stress with Age, Gender, and Employment Length as Covariates**

***Depression***

| Model Fit Measures | | | | | | | |
| --- | --- | --- | --- | --- | --- | --- | --- |
|  | | | | Overall Model Test | | | |
| Model | R | R² | Adjusted R² | F | df1 | df2 | p |
| 1 | 0.39718 | 0.15775 | 0.09697 | 2.59535 | 7 | 97 | .0169 |

| Omnibus ANOVA Test | | | | | |
| --- | --- | --- | --- | --- | --- |
|  | Sum of Squares | df | Mean Square | F | p |
| High Arousal | 119.55505 | 1 | 119.55505 | 1.70927 | .1942 |
| Low Arousal | 0.29565 | 1 | 0.29565 | 0.00423 | .9483 |
| Positive Valence | 229.66381 | 1 | 229.66381 | 3.28349 | .0731 |
| Negative Valence | 901.09972 | 1 | 901.09972 | 12.88297 | .0005 |
| Gender | 91.72736 | 1 | 91.72736 | 1.31142 | .2550 |
| Age | 111.62625 | 1 | 111.62625 | 1.59591 | .2095 |
| Employment Length | 185.82520 | 1 | 185.82520 | 2.65673 | .1064 |
| Residuals | 6784.66624 | 97 | 69.94501 |  |  |

| Model Coefficients - Depression | | | | | |
| --- | --- | --- | --- | --- | --- |
| Predictor | Estimate | SE | t | p | Stand. Estimate |
| Interceptᵃ | 4.89090 | 4.22597 | 1.15734 | .2500 |  |
| High Arousal | -0.51592 | 0.39462 | -1.30739 | .1942 | -0.14973 |
| Low Arousal | -0.01973 | 0.30348 | -0.06501 | .9483 | -0.00614 |
| Positive Valence | 0.43742 | 0.24140 | 1.81204 | .0731 | 0.17422 |
| Negative Valence | 1.08839 | 0.30323 | 3.58929 | .0005 | 0.40236 |
| Gender: |  |  |  |  |  |
| Male – Female | 2.29797 | 2.00666 | 1.14517 | .2550 | 0.26111 |
| Age | -0.11752 | 0.09303 | -1.26330 | .2095 | -0.14438 |
| Employment Length | 0.01894 | 0.01162 | 1.62995 | .1064 | 0.18829 |

***Anxiety***

| Model Fit Measures | | | | | | | |
| --- | --- | --- | --- | --- | --- | --- | --- |
|  | | | | Overall Model Test | | | |
| Model | R | R² | Adjusted R² | F | df1 | df2 | p |
| 1 | 0.41729 | 0.17413 | 0.11453 | 2.92173 | 7 | 97 | .0081 |

| Omnibus ANOVA Test | | | | | |
| --- | --- | --- | --- | --- | --- |
|  | Sum of Squares | df | Mean Square | F | p |
| High Arousal | 41.21295 | 1 | 41.21295 | 0.93515 | .3359 |
| Low Arousal | 21.74923 | 1 | 21.74923 | 0.49350 | .4841 |
| Positive Valence | 54.47523 | 1 | 54.47523 | 1.23608 | .2690 |
| Negative Valence | 593.93717 | 1 | 593.93717 | 13.47683 | .0004 |
| Gender | 49.52996 | 1 | 49.52996 | 1.12387 | .2917 |
| Age | 111.81952 | 1 | 111.81952 | 2.53726 | .1144 |
| Employment Length | 154.77338 | 1 | 154.77338 | 3.51191 | .0639 |
| Residuals | 4274.88502 | 97 | 44.07098 |  |  |

| Model Coefficients - Anxiety | | | | | |
| --- | --- | --- | --- | --- | --- |
| Predictor | Estimate | SE | t | p | Stand. Estimate |
| Interceptᵃ | 5.39640 | 3.35448 | 1.60871 | .1109 |  |
| High Arousal | -0.30291 | 0.31324 | -0.96703 | .3359 | -0.10967 |
| Low Arousal | 0.16923 | 0.24090 | 0.70250 | .4841 | 0.06572 |
| Positive Valence | 0.21303 | 0.19161 | 1.11179 | .2690 | 0.10585 |
| Negative Valence | 0.88363 | 0.24070 | 3.67108 | .0004 | 0.40751 |
| Gender: |  |  |  |  |  |
| Male – Female | -1.68861 | 1.59284 | -1.06013 | .2917 | -0.23935 |
| Age | -0.11762 | 0.07384 | -1.59288 | .1144 | -0.18026 |
| Employment Length | 0.01728 | 0.00922 | 1.87401 | .0639 | 0.21436 |

***Stress***

| Model Fit Measures | | | | | | | |
| --- | --- | --- | --- | --- | --- | --- | --- |
|  | | | | Overall Model Test | | | |
| Model | R | R² | Adjusted R² | F | df1 | df2 | p |
| 1 | 0.27953 | 0.07814 | 0.01161 | 1.17453 | 7 | 97 | .3245 |

| Omnibus ANOVA Test | | | | | |
| --- | --- | --- | --- | --- | --- |
|  | Sum of Squares | df | Mean Square | F | p |
| High Arousal | 85.94905 | 1 | 85.94905 | 1.17459 | .2811 |
| Low Arousal | 34.84902 | 1 | 34.84902 | 0.47625 | .4918 |
| Positive Valence | 136.48653 | 1 | 136.48653 | 1.86525 | .1752 |
| Negative Valence | 233.54088 | 1 | 233.54088 | 3.19161 | .0771 |
| Gender | 112.83174 | 1 | 112.83174 | 1.54198 | .2173 |
| Age | 103.48703 | 1 | 103.48703 | 1.41427 | .2373 |
| Employment Length | 98.53233 | 1 | 98.53233 | 1.34656 | .2487 |
| Residuals | 7097.82012 | 97 | 73.17340 |  |  |

| Model Coefficients - Stress | | | | | |
| --- | --- | --- | --- | --- | --- |
| Predictor | Estimate | SE | t | p | Stand. Estimate |
| Interceptᵃ | 11.72931 | 4.32240 | 2.71361 | .0079 |  |
| High Arousal | -0.43744 | 0.40362 | -1.08379 | .2811 | -0.12986 |
| Low Arousal | 0.21421 | 0.31040 | 0.69011 | .4918 | 0.06821 |
| Positive Valence | 0.33721 | 0.24690 | 1.36574 | .1752 | 0.13737 |
| Negative Valence | 0.55409 | 0.31015 | 1.78651 | .0771 | 0.20952 |
| Gender: |  |  |  |  |  |
| Male – Female | -2.54866 | 2.05245 | -1.24176 | .2173 | -0.29621 |
| Age | -0.11315 | 0.09515 | -1.18923 | .2373 | -0.14219 |
| Employment Length | 0.01379 | 0.01188 | 1.16041 | .2487 | 0.14024 |
